# Determining the geographical origin of common buckwheat from China by multivariate analysis based on mineral elements, amino acids and vitamins

**DOI:** 10.1038/s41598-017-08808-y

**Published:** 2017-08-29

**Authors:** Qiang Zhang, Jian-Guo Xu

**Affiliations:** 1 School of Food Science, Shanxi Normal University, Linfen City, 041004 China; 2 School of Life Sciences, Shanxi Normal University, Linfen City, 041004 China

## Abstract

This study aimed to establish a method for distinguishing the geographical origin of common buckwheat from Inner Mongolia, Shanxi and Shaanxi Provinces in China. Three chemical families including mineral elements, vitamins and amino acids of 48 samples from different geographical origins were analyzed by principal component analysis (PCA), cluster analysis (CA) and linear discriminate analysis (LDA) for this purpose. LDA clearly discriminated the geographical origin of common buckwheat samples grown in three regions, and gave a high correct classification rate of 95.8% and satisfactory cross-validation rate of 91.7%. Some variables (Mn, VPP, Se, Gly, Cu, Asp, Fe, and Ala) significantly contributed to the ability to discriminate the geographical origin of the common buckwheat. These results demonstrated that the proposed method is a powerful tool for controlling the geographical origin of common buckwheat by governmental administration and protecting consumers from improper domestic labeling. However, the discriminant method still needs to be further validated using more reliable data.

## Introduction

Nutrition and the biological activity components from plant-derived products are greatly influenced by geographic origins because of differences in weather conditions, geographic area, and soil, which resulted in change of the quality and prices of agricultural products^1, 2^. In addition, the loss of identity of food origin may expose consumers to many risks derived by the cultivation processes due to the market globalization and consequently to the easier circulation of foodstuffs^3^. For these reasons, determining the geographic origin of agricultural food has become a field of increasing importance for consumers. Currently, there have been numerous efforts and methods including multielement, organic compounds, physicochemical parameters analysis combined with chemometrics or multivariate data analysis to certify the geographical origin of food and plant products^4–7^.

Common buckwheat (*Fagopyrum esculentum* Moench) contains a variety of nutrients and bioactive phytochemicals^8–10^, and is therefore not only an important source of basic nutrition, but may also provide other positive health benefits^11–13^. The consumption of this product has become increasingly popular in the United States, Canada, and Europe. Common buckwheat production is worldwide concentrated in China, which is the biggest world producer generally^14^. Especially, Inner Mongolia, Shanxi and Shaanxi Provinces are the major common buckwheat production areas in China. However, common buckwheat is an obligate cross pollinating crop because of its sporophytic self-incompatibility system^15^, i.e., we cannot get relatively genetic pure seeds in any generation, together with stable morphological traits using this kind of seeds. Studies on common buckwheat mainly focused on morphological and ecological characteristics, variety selection and cultivation as well as nutritional ingredients, but there have been few reports of its geographical origin traceability^8–12, 14, 16^.

In the present study, the characteristics of some mineral elements, vitamins and amino acids in common buckwheat cultivated from Inner Mongolia, Shanxi and Shaanxi regions are analyzed and compared, and further determined the geographical origin of common buckwheat based on multivariate analysis. The aim of this study is to provide efficient method for distinguishing the geographical origins of common buckwheat from different regions, which is of great importance for the quality control and food authenticity of common buckwheat.

## Results

### Elemental profiles

The seven elements concentrations (Cu, Zn, Fe, Mn, Ca, P and Se) of 48 common buckwheat samples from Inner Mongolia, Shanxi and Shaanxi are shown in Table 1. There is no significant difference in the mean concentration of Zn, Fe, Ca and P among the Inner Mongolia, Shanxi and Shaanxi (p > 0.05) which are rejected for further statistical analysis, while Cu, Mn and Se in samples were significantly different among the regions. Inner Mongolia samples could be clearly separated from Shanxi and Shaanxi samples based on the highest content of Mn and the lowest content of Cu and Se. Shanxi and Shaanxi samples had the highest content of Se and Cu, respectively, but there was no significant difference between Shanxi and Shaanxi samples.

**Table 1:** Descriptive statistics for mineral content (μg/g) and vitamin content (mg/100 g) in common buckwheat of different regions.

	Inner Mongolia (n = 21)	Shanxi (n = 19)	Shaanxi (n = 8)	p-value
Mineral				
Cu	9.43 ± 1.57 a	11.63 ± 0.45 b	16.22 ± 4.75 b	0.002
Zn	21.14 ± 1.26 a	24.44 ± 2.03 a	24.29 ± 2.98 a	0.320
Fe	149.39 ± 34.31 a	118.17 ± 18.15 a	130.49 ± 28.48 a	0.683
Mn	18.49 ± 0.93 b	12.96 ± 0.72 a	12.22 ± 0.92 a	0.000
Ca	497 ± 149.19 a	465 ± 58.03 a	504 ± 83.89 a	0.227
P	3373 ± 165.20 a	3258 ± 276.94 a	3953 ± 278.72 a	0.099
Se	0.030 ± 0.003 a	0.090 ± 0.017 b	0.06 ± 0.01 b	0.003
Vitamin				
VE	0.95 ± 0.13 a	1.64 ± 0.24 b	1.38 ± 0.18 ab	0.029
VPP	3.17 ± 0.29 a	4.47 ± 0.28 b	2.08 ± 0.35 a	0.000

Values represent means of their different independent replicates ± SE, respectively. Different letters within a row indicate statistically significant differences between the means (p < 0.05).

### Profiles of vitamin E and vitamin PP

As seen in Table 1, Vitamin PP and vitamin E in samples were significantly different among the different regions. The common buckwheat from Shanxi had the highest content of vitamin PP (4.47 mg/100 g) and vitamin E (1.64 mg/100 g), while the lowest content of vitamin E (0.95 mg/100 g) and vitamin PP (2.08 mg/100 g) were found in Inner Mongolia and Shaanxi samples, respectively.

### Profiles of amino acids

The characteristics of amino acids in common buckwheat from different regions are presented in Table 2. There was significant difference in the mean content of Asp, Glu, Gly, Ala, Met and Lys among the Inner Mongolia, Shanxi and Shaanxi (p < 0.05), while no obvious difference was found in other amino acids from samples. The significant difference in amino acids concentrations of common buckwheat samples made it possible to distinguish them from different regions and provided reliable results for further statistical analysis.

**Table 2:** Descriptive statistics for amino acids content (g/100 g) in common buckwheat of different regions.

Amino acids	Inner Mongolia (n = 21)	Shanxi (n = 19)	Shaanxi (n = 8)	p-value
Asp	0.99 ± 0.04 a	1.15 ± 0.07 b	1.16 ± 0.06 b	0.046
Thr	0.40 ± 0.02 a	0.45 ± 0.02 a	0.45 ± 0.02 a	0.074
Ser	0.52 ± 0.02 a	0.59 ± 0.03 a	0.60 ± 0.03 a	0.059
Glu	1.97 ± 0.08 a	2.28 ± 0.15 ab	2.39 ± 0.12 b	0.032
Gly	0.59 ± 0.02 a	0.64 ± 0.03 ab	0.71 ± 0.02 b	0.014
Ala	0.45 ± 0.02 a	0.51 ± 0.02 b	0.54 ± 0.02 b	0.014
Cys	0.15 ± 0.01 a	0.16 ± 0.02 a	0.18 ± 0.01 a	0.189
Val	0.51 ± 0.02 a	0.56 ± 0.03 a	0.61 ± 0.03 a	0.108
Met	0.15 ± 0.01 a	0.19 ± 0.02 b	0.19 ± 0.01 b	0.023
Ile	0.45 ± 0.02 a	0.46 ± 0.03 a	0.49 ± 0.03 a	0.285
Leu	0.70 ± 0.02 a	0.78 ± 0.04 a	0.80 ± 0.04 a	0.123
Tyr	0.30 ± 0.01 a	0.30 ± 0.02 a	0.32 ± 0.01 a	0.330
Phe	0.51 ± 0.02 a	0.54 ± 0.02 a	0.58 ± 0.03 a	0.141
Lys	0.59 ± 0.02 a	0.65 ± 0.03 ab	0.71 ± 0.03 b	0.019
His	0.24 ± 0.01 a	0.26 ± 0.01 a	0.28 ± 0.01 a	0.115
Arg	1.00 ± 0.05 a	1.07 ± 0.06 a	1.15 ± 0.08 a	0.237
Pro	0.41 ± 0.02 a	0.45 ± 0.02 a	0.46 ± 0.03 a	0.186
Trp	0.12 ± 0.00 a	0.12 ± 0.01 a	0.11 ± 0.01 a	0.638
Total	10.04 ± 0.39 a	11.15 ± 0.62 a	11.70 ± 0.54 a	0.090

Values represent means of their different independent replicates ± SE, respectively. Different letters within a row indicate statistically significant differences between the means (p < 0.05).

### Principal component analysis (PCA)

In order to evaluate the difference of common buckwheat from different regions, indicators with significant differences (p < 0.05) was respectively processed by PCA. Table 3 showed the results of PCA and their discriminant analysis. The correct classification rate and their cross-validation rate of both model 1 (based on vitamin content), model 2 (based on mineral element content) and model 3 (based on amino acid content) were no more than 50%. The highest correct classification rate (79.2%) and their cross-validation rate (79.2%) were found in model 5 based on the combination of the content of amino acid, mineral element and vitamin content as well as relative content of amino acid. The discriminant results based on PCA were difficult to distinguish common buckwheat origins. Therefore, other statistical analysis methods should be further employed to obtain better results.

**Table 3:** Discrimination model based on PCA and their accuracy.

Model No.	Variable types	Amount of PC	Correct classification rate (%)	Cross-validation rate (%)
1	vitamin	1	37.5	37.5
2	mineral element	2	50.0	41.7
3	amino acid content	2	50.0	43.8
4	relative content of amino acid	6	64.6	62.5
5	mineral element + vitamin + content and relative content of amino acid	10	79.2	79.2

### Cluster analysis (CA)

**Figure 1:**
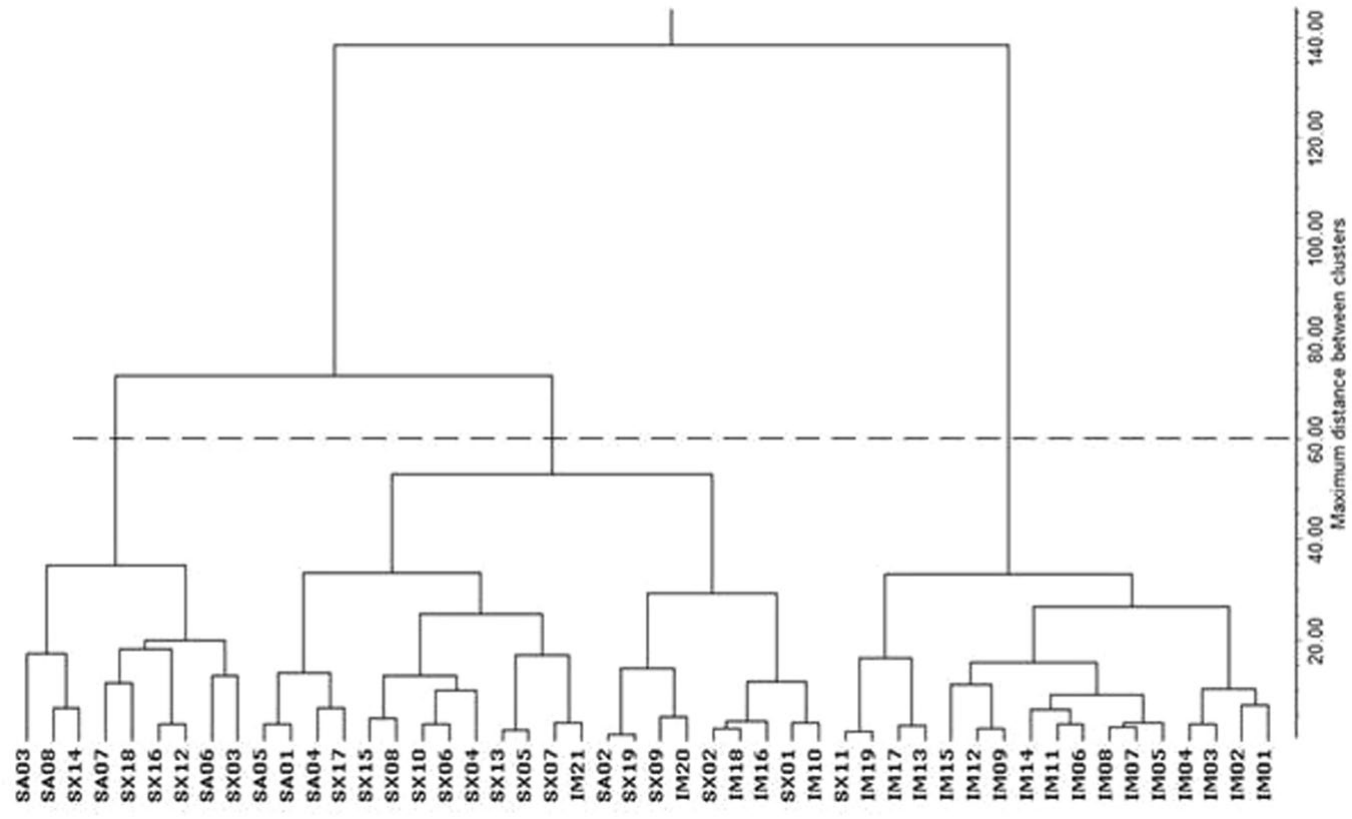
Dendrogram of cluster analysis.

To better visualize the relative distribution of the common buckwheat, CA was performed according to variables with significant differences (p < 0.05). The samples were grouped into clusters in terms of their nearness or similarity which was measured based on the Mahalanobis distance. The smallest distance indicated the highest degree of relationship, therefore, those objects are considered to belong to the same group. All samples from different regions were separated into three clusters based on the dendrogram cut at a distance of 60 (Fig. 1). The first cluster was composed of Shaanxi (n = 4) and Shanxi (n = 5). The second cluster was composed of samples from Inner Mongolia (n = 5), Shaanxi (n = 4) and Shanxi (n = 13), and the third cluster was composed of Inner Mongolia (n = 16) and only one Shanxi sample. The results indicated that CA could give a rough location distribution, but not well determined the geographical origin of common buckwheat, which was consistent with the results from PCA. Obviously, the use of PCA and CA in combination with all variables did not enable a good discrimination of the geographical origin of common buckwheat.

### Linear discriminant analysis (LDA)

For achieving better classification and identification of the common buckwheat samples from different regions, the stepwise discriminant procedure was carried out to extract best discriminant variable separating samples from different origins, which entered or removed variables by analyzing their effects on the discrimination of the groups based on the Wilks’ lambda criterion. Table 4 summarized the observation of the cross-validation results together with the classification of common buckwheat samples using LDA model. The correct classification rate of model 1 (based on amino acid content), model 2 (based on vitamin content), model 3 (based on mineral element content) and model 4 (based on relative content of amino acid) were 60.4%, 62.5%, 66.7%, 77.1% and their cross-validation rate reached to 54.2%, 60.4%, 62.5%, and 72.9% respectively, which indicated that mineral elements, amino acids and vitamins compositions of common buckwheat from different origins was similar, making it difficult to distinguish the origins using one variable alone. In model 5, the combination of mineral element, vitamin and amino acid content as well as relative content of amino acid, was taken as the variable, the correct classification rate and cross-validation rate reached 95.8% and 91.7%, respectively.

**Table 4:** Observations of the cross-validation results and discrimination model.

Model No.	Variable types	Effective indicators	Correct rate (%)	Cross-validation rate (%)
1	amino acid content	Met	60.4	54.2
2	vitamin	VPP, VE	62.5	60.4
3	mineral element	Cu, Mn, Se	66.7	62.5
4	relative content of amino acid	Asp, Met, Leu, Tyr	77.1	72.9
5	mineral element + vitamin + content and relative content of amino acid	Mn, VPP, Se, Gly, Cu, Asp, Fe, Ala, relative content of Ala	95.8	91.7

**Figure 2:**
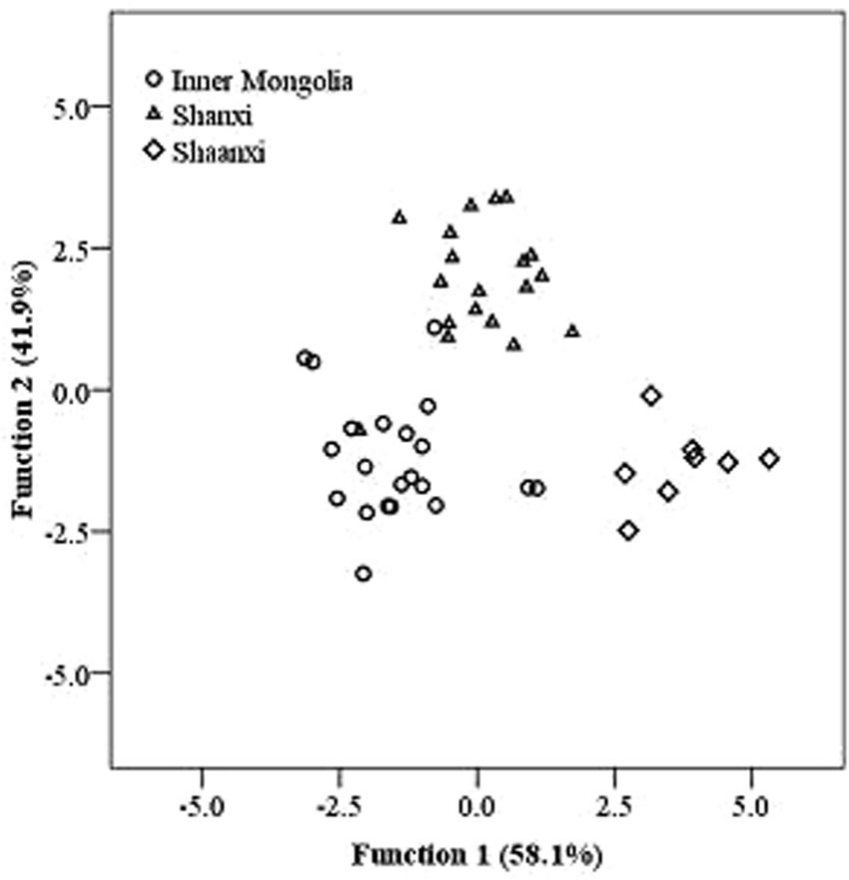
Scatter plot of common buckwheat from different regions based on the two discriminant functions.

In model 5, nine variables (content of Mn, Se, Cu, Fe, VPP, Gly, Asp and Ala as well as relative content of Ala) were selected and thought to contribute significantly to the ability for discriminating the geographical origin (Table 4), and two discriminant functions were constructed on the basis of Wilks’ lambda values (Fig. 2). The two functions explained the 100% of the variance (Function 1 explained 58.1% of the total variance, and function 2 explained 41.9%). Discriminant functions are shown as follows,

Function 1 = −7.557–14.603Asp + 23.939Gly + 12.366Ala + 99.904Ala (relative content) + 0.219Cu–0.008Fe–0.183Mn + 7.281Se–0.360Vpp.

Function 2 = −26.094 + 20.134Asp–6.402Gly–43.015Ala + 612.306Ala (relative content) – 0.001Cu + 0.001Fe−0.081Mn + 14.083Se + 0.493Vpp.

The separation of common buckwheat from Inner Mongolia, Shanxi and Shaanxi was checked by plotting the two functions scores (Fig. 2). It is clearly shown that common buckwheat from different regions was well distinguished from each other, confirming that selected variables provided the useful information for common buckwheat classification. To evaluate the predictive capacity, the generated model was then validated by the leave-one out cross-validation method and the LDA classification results of model 5 are summarized in Table 5. According to the selected nine indicators, the correct classification rate reached 95.2%, 94.7% and 100% for common buckwheat from Inner Mongolia, Shanxi and Shaanxi, respectively. The predictive ability of this model was 91.7%, indicating a satisfactory performance of this model for the classification of common buckwheat samples from different origins. These results indicated that the LDA method can effectively distinguish the common buckwheat from different origins.

**Table 5:** Classification of common buckwheat in different regions and percentage of observations correctly classified by LDA.

			Predicted group membership			
			IM^a^	SX^b^	SA^c^	Total
Original	Count	IM	20	1	0	21
		SX	1	18	0	19
		SA	0	0	8	8
	%		95.2	94.7	100	95.8
Cross-validated	Count	IM	18	1	2	21
		SX	1	18	0	19
		SA	0	0	8	8
	%		85.7	94.7	100	91.7

^a^IM, Inner Mongolia; ^b^SX, Shanxi; ^c^SA, Shaanxi.

## Discussion

The characteristics of plant-derived products can be highly influenced by several environmental and geological factors such as soil type, soil parent material, water, soil pH, and climate conditions. The element analysis is usually considered to be an effective tool, because plants can absorb the mineral elements from the soil and thus there is an association to some extent between the contents of mineral elements in environment and their accumulation degree in crops^17, 18^. The method of element analysis has been applied for geographical origin assignment of some farm products such as wine^19^, honey^20^, mutton^21^, sheep milk^22, 23^, Chinese cabbage^24, 25^, tea^6^, coffee^4^, wheat^26, 27^, and other crops^28^ as well as some aquatic products^29, 30^ with different degrees of success. Besides, some organic compounds or physicochemical parameters (color, diastase activity, electrical conductivity, total antioxidant activity, etc.) have also been used to determine the geographical origin of some food and agricultural products^23, 31–33^. Amino acids are important components of foods, and they contributed directly to the taste of foods and color when heating foods. Some studies determined successfully the geographic origin of some agricultural products based on amino acids analysis^1, 34, 35^. In recent years, multivariate geographical origin traceability and discrimination study by combinations of various types of substances has been used in the field of agricultural product in order to avoid the one-sidedness of variation of a kind of constituent^32, 33, 36, 37^.

In the present study, discriminant analysis based on PCA and CA did not well determine the geographical origin of common buckwheat. Similarly, LDA using the stepwise method did not well determine the geographical origin when only a chemical family was analyzed independently. However, LDA method can effectively distinguish the common buckwheat from different origins based on the combination of three chemical families (mineral element, vitamin and amino acid), and the correct classification rate and cross-validation rate reached 95.8% and 91.7%, respectively.

Inner Mongolia Province covered a vast geographic area, with different soil types from east to west, such as dark brown soil, chestnut soil, brown loam soil, sand land and gray brown desert soil. Inner Mongolia is located in high latitude, and the area was a temperate continental monsoon climate. The Shanxi Plateau belonged to the warm temperate zone and temperate continental climate, with the complicated topography, loessal soils and brown soils. The differences of temperature and climate conditions were obvious because of longer distance of Shanxi Plateau from north to south. The Shaanxi Plateau was located in the transitional zone between China’s southeast humid region and the northwest arid region, and was mainly in the continental middle temperate zone and the soil mainly made of loessal soils. These differences provided the feasibility for distinguishing the geographic origin of common buckwheat from Inner Mongolia, Shanxi and Shaanxi Provinces.

## Conclusion

In summary, the present study showed that LDA using the stepwise method was much more effective than PCA and CA for classification of geographic origin of common buckwheat from Inner Mongolia, Shanxi and Shaanxi Provinces based on the combining the mineral element, vitamin and amino acid compositions. As suggested by LDA, some variables (Mn, Se, Cu, Fe, VPP, Gly, Asp and Ala) were regarded as the good classifier for determining geographical origin of common buckwheat, and the correct classification rate and cross-validation rate reached 95.8% and 91.7%, respectively. Therefore, the results of this study can provide theoretical data and also be used as a powerful recognition tool for the origin traceability and identification of common buckwheat. However, LDA discriminant method still needs to be further validated using more reliable data.

## Methods

### Data sources

**Figure 3:**
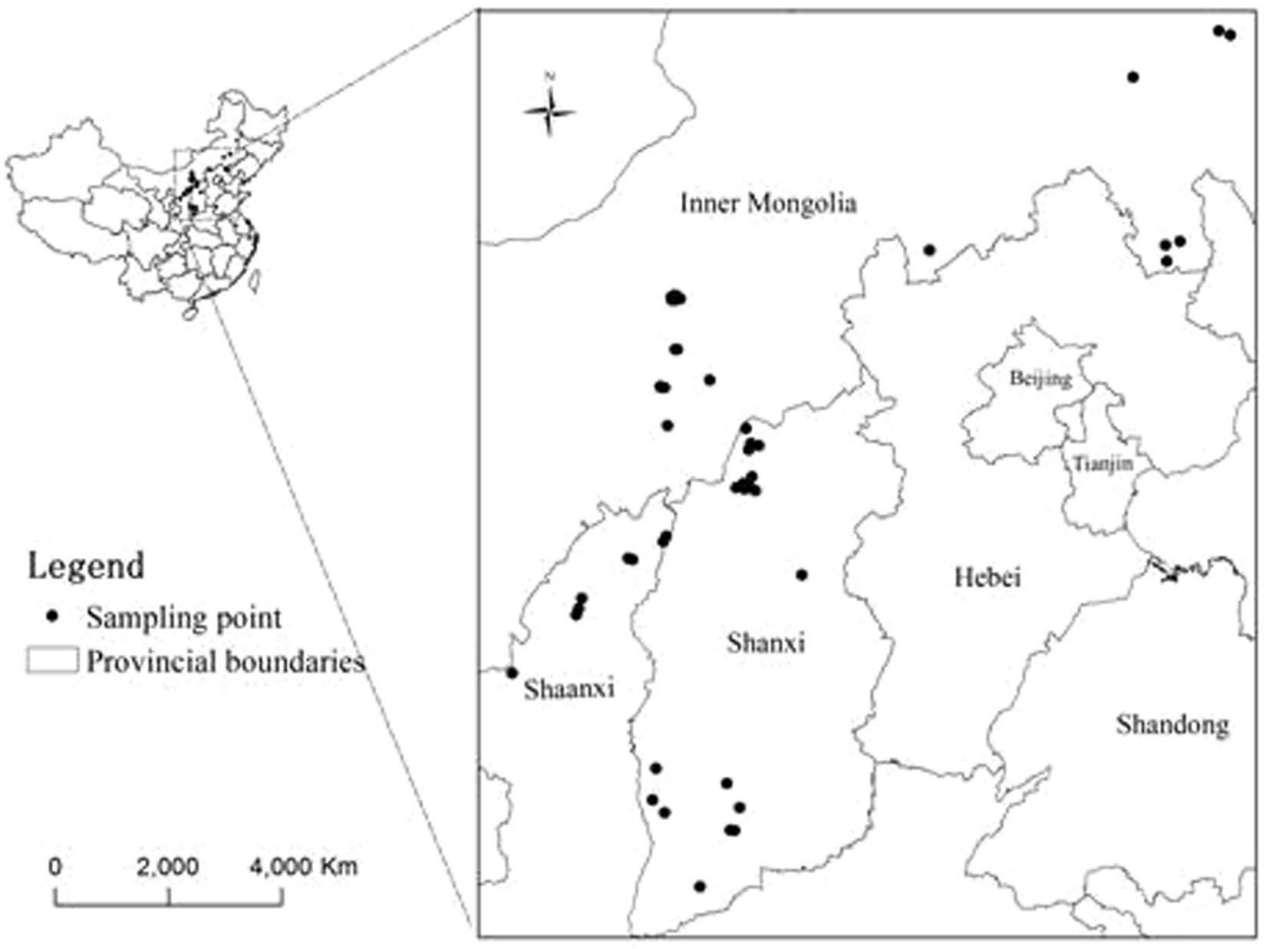
Geographical origins of the common buckwheat samples. This map was generated by ArcGIS software (version 9.2, http://www.esri.com).

Data of mineral elements, vitamins and amino acids in common buckwheat were collected from Chinese Crop Germplasm Resources Information System (CGRIS) which provides data for the public (http://icgr.caas.net.cn). Complete data of 48 common buckwheat samples cultivated in Inner Mongolia, Shanxi and Shaanxi Provinces which are the main production regions of common buckwheat in China were obtained from the database. The content of Cu, Mn, Fe, Zn, and Ca were determined by atomic absorption method; the content of Se and P were determined by hydride atomic fluorescence spectrometry and spectrophotometric methods, respectively; the content of amino acids were determined using Amino Acid Analyzer; the content of VPP and VE were determined by gas chromatography and photocolorimetric methods, respectively. The locations and details of samples are shown in Fig. 3 and Table 6.

**Table 6:** Information of common buckwheat samples.

No.	Unified code in CGRIS	Site	No.	Unified code in CGRIS	Site
IM01	00000115	Wuchuan, Inner Mongolia	SX04	00000339	Youyu, Shanxi
IM02	00000116	Wuchuan, Inner Mongolia	SX05	00000340	Pinglu, Shanxi
IM03	00000212	Taipusi, Inner Mongolia	SX06	00000341	Pinglu, Shanxi
IM04	00000213	Damao, Inner Mongolia	SX07	00000342	Pinglu, Shanxi
IM05	00000214	Damao, Inner Mongolia	SX08	00000344	Pinglu, Shanxi
IM06	00000215	Damao, Inner Mongolia	SX09	00000345	Pinglu, Shanxi
IM07	00000216	Damao, Inner Mongolia	SX10	00000346	Pinglu, Shanxi
IM08	00000217	Damao, Inner Mongolia	SX11	00000349	Dingxiang, Shanxi
IM09	00000218	Damao, Inner Mongolia	SX12	00000429	Hongtong, Shanxi
IM10	00000219	Damao, Inner Mongolia	SX13	00000430	Fushan, Shanxi
IM11	00000220	Tuzuo, Inner Mongolia	SX14	00000433	Yicheng, Shanxi
IM12	00000221	Tuzuo, Inner Mongolia	SX15	00000434	Yicheng, Shanxi
IM13	00000222	Tuzuo, Inner Mongolia	SX16	00000435	Daning, Shanxi
IM14	00000223	Tuoxian, Inner Mongolia	SX17	00000437	Jixian, Shanxi
IM15	00000224	Huhehaote, Inner Mongolia	SX18	00000439	Xiangning, Shanxi
IM16	00000225	Ningcheng, Inner Mongolia	SX19	00000443	Xiaxian, Shanxi
IM17	00000226	Ningcheng, Inner Mongolia	SA01	00000477	Fugu, Shaanxi
IM18	00000227	Ningcheng, Inner Mongolia	SA02	00000478	Fugu, Shaanxi
IM19	00000228	Balinyou, Inner Mongolia	SA03	00000479	Shenmu, Shaanxi
IM20	00000229	Aluke, Inner Mongolia	SA04	00000480	Shenmu, Shaanxi
IM21	00000230	Aluke, Inner Mongolia	SA05	00000481	Yulin, Shaanxi
SX01	00000334	Youyu, Shanxi	SA06	00000482	Yulin, Shaanxi
SX02	00000335	Youyu, Shanxi	SA07	00000483	Yulin, Shaanxi
SX03	00000337	Youyu, Shanxi	SA08	00000484	Jingbian, Shaanxi

### Statistical analyses

Analysis of variance was first carried on each single component of all the samples to determine significant differences (*p* < 0.05). Unsupervised classification was performed with cluster analysis (CA) to measure the similarity between samples, and CA was carried out by DPS 16.05 software based on standardization transformation of data, Mahalanobis distance and flexible group average method. Principal component analysis (PCA) was used to reduce the dimensionality of the data for linear data analysis, and the extraction of principal component was based on the eigenvalue greater than 1. Linear discriminant analysis (LDA) using the stepwise method was carried out to evaluate whether samples from different regions could be mathematically distinguished. The statistical significance of each discriminant function was evaluated on the basis of the Wilks’ lambda and F value criteria, and predictive ability of classification model was evaluated by a cross-validation test, using the ‘leave-one-out’ procedure. Analysis of variance, PCA and LDA were performed by the IBM SPSS Statistics 19 package for windows.
